# [1,2-Bis(diphenyl­phosphino)ethane-κ^2^ *P*,*P*′](2-carboxyl­atothio­phenolato-κ^2^ *O*,*S*)nickel(II) methanol solvate

**DOI:** 10.1107/S1600536808044280

**Published:** 2009-01-08

**Authors:** Jinling Miao, Furong Li, Haiyan Chen, Daqi Wang, Yong Nie

**Affiliations:** a School of Chemistry and Chemical Engineering, University of Jinan, Jinan 250022, People’s Republic of China; b College of Chemistry and Chemical Engineering, Liaocheng University, Liaocheng 252059, People’s Republic of China

## Abstract

In the title complex, [Ni(C_7_H_4_O_2_S)(C_26_H_24_P_2_)]·CH_3_OH, the nickel(II) centre adopts an approximately square-planar geometry, with the Ni atom coordinating to the S and O atoms of the bidentate thio­salicylate ligand and the two P atoms of the chelating Ph_2_PCH_2_CH_2_PPh_2_ ligand. There is hydrogen bonding between the methanol solvent mol­ecule and the carbonyl O atom of the thio­salicylate ligand.

## Related literature

For previous preparations and structures of the non-solvated complex, see: Kang *et al.* (1998 ▶); McCaffrey *et al.* (1997 ▶).
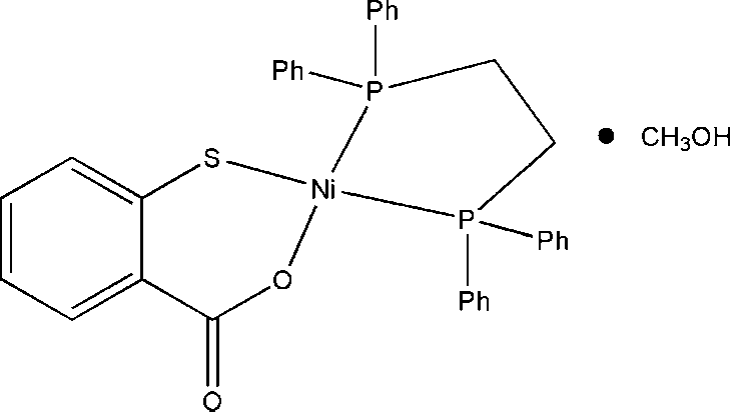


## Experimental

### 

#### Crystal data

[Ni(C_7_H_4_O_2_S)(C_26_H_24_P_2_)]·CH_4_O
*M* *_r_* = 641.31
Monoclinic, 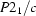

*a* = 13.9229 (15) Å
*b* = 11.6244 (10) Å
*c* = 19.553 (2) Å
β = 100.085 (2)°
*V* = 3115.6 (6) Å^3^
*Z* = 4
Mo *K*α radiation
μ = 0.83 mm^−1^
*T* = 293 (2) K
0.48 × 0.37 × 0.32 mm

#### Data collection

Bruker SMART 1000 CCD area-detector diffractometer
Absorption correction: multi-scan (*SADABS*; Bruker, 2001 ▶) *T*_min_ = 0.693, *T*_max_ = 0.778
14599 measured reflections
5484 independent reflections
3538 reflections with *I* > 2σ(*I*)
*R*_int_ = 0.032

#### Refinement

*R*[*F* ^2^ > 2σ(*F* ^2^)] = 0.042
*wR*(*F* ^2^) = 0.117
*S* = 1.10
5484 reflections
370 parameters
H-atom parameters constrained
Δρ_max_ = 0.42 e Å^−3^
Δρ_min_ = −0.26 e Å^−3^

### 

Data collection: *SMART* (Bruker, 2001 ▶); cell refinement: *SAINT* (Bruker, 2001 ▶); data reduction: *SAINT*; program(s) used to solve structure: *SHELXS97* (Sheldrick, 2008 ▶); program(s) used to refine structure: *SHELXL97* (Sheldrick, 2008 ▶); molecular graphics: *SHELXTL* (Sheldrick, 2008 ▶); software used to prepare material for publication: *SHELXTL*.

## Supplementary Material

Crystal structure: contains datablocks I, global. DOI: 10.1107/S1600536808044280/sj2555sup1.cif

Structure factors: contains datablocks I. DOI: 10.1107/S1600536808044280/sj2555Isup2.hkl

Additional supplementary materials:  crystallographic information; 3D view; checkCIF report

## Figures and Tables

**Table:** 

Ni1—O1	1.905 (3)
Ni1—P1	2.1378 (11)
Ni1—S1	2.1775 (11)
Ni1—P2	2.2114 (10)

**Table:** 

O1—Ni1—P1	178.81 (8)
O1—Ni1—S1	93.94 (8)
P1—Ni1—S1	86.56 (4)
O1—Ni1—P2	92.22 (8)
P1—Ni1—P2	87.20 (4)
S1—Ni1—P2	172.70 (4)

**Table 2:** Hydrogen-bond geometry (Å, °)

*D*—H⋯*A*	*D*—H	H⋯*A*	*D*⋯*A*	*D*—H⋯*A*
O3—H3⋯O2^i^	0.82	1.88	2.697 (5)	171

Symmetry code: (i) 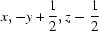.

**Fig. 1.**
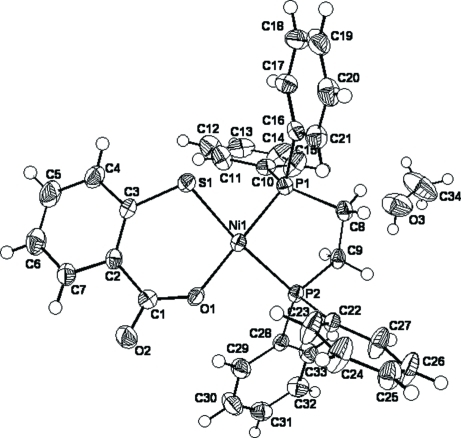
The molecular structure, with atom labels and 25% probability displacement ellipsoids for non-H atoms.

## supplementary crystallographic information

### Comment

The synthesis and crystal structure of [(dppe)Ni(tsal)] (where dppe is Ph_2_PCH_2_CH_2_PPh_2_ and tsal is thiosalicylato) have been described by McCaffrey *et al.* (1997) by a reaction of NiCl_2_(dppe) and tsalH_2_ in the presence of pyridine, and also by Kang *et al.* (1998) *via* a similar reaction of NiCl_2_, dppe and thiosalicylate. We have recently obtained the same complex as a methanol solvate when NiCl_2_(dppe) was reacted with thiosalicylic acid in the presence of NaOH as a base.

As shown in Fig. 1, the coordination geometry around the nickel center is approximately square planar. The sum of the bond angles around the Ni atom is 359.92°, with the *trans* P—Ni—S and P—Ni—O angles being 172.70 and 178.81°, respectively, Table 1, while in related structures the corresponding values were found to be 361.45, 170.99 and 170.73° (McCaffrey *et al.*, 1997), and 358.4, 166.0, 173.0°, (Kang *et al.*, 1998) respectively. These indicate that in the present structure the P_2_OS unit is slightly more planar. As expected, the Ni1—P2 bond length (opposite to S, 2.2114 (10) Å) is found to be longer than that for Ni1—P1 (opposite to O, 2.1378 (11) Å), due to the different *trans* influence of the S and O atoms. Strong O3—H3···O2^i^ [i = *x*, -*y* + 1/2, *z* - 1/2] hydrogen bonding (2.697 Å) is observed between the methanol solvate molecule and the carbonyl O atom of the thiosalicylato group, Table 2.

### Experimental

Thiosalicylic acid (32 mg, 0.2 mmol) was added to a solution of NaOH (0.2 mmol) in methanol (2 ml) to give a slightly yellow solution. This was transferred dropwise to a suspension of NiCl_2_(dppe) (53 mg, 0.1 mmol) in CH_3_CN (3 ml). After stirring for 10 min, a deep-red solution formed, from which red crystals (55 mg, 85%) were grown on standing at room temperature. IR (KBr): ν = 3399, 3053, 1596, 1435, 1351, 1102, 746, 690, 531 cm ^-1^.

### Refinement

All H atoms were positioned geometrically and refined using a riding model with d(C—H) = 0.93 Å, U_iso_ = 1.2U_eq_(C) for aromatic, 0.97 Å, U_iso_ = 1.2U_eq_(C) for CH_2_, 0.96 Å, U_iso_ = 1.5U_eq_(C) for CH_3_ atoms and 0.82 Å, U_iso_ = 1.5U_eq_(O) for the OH groups.

### Crystal data

**Table:**

[Ni(C_7_H_4_O_2_S)(C_26_H_24_P_2_)]·CH_4_O	*F*(000) = 1336
*M**_r_* = 641.31	*D*_x_ = 1.367 Mg m^−^^3^
Monoclinic, *P*2_1_/*c*	Mo *K*α radiation, λ = 0.71073 Å
Hall symbol: -P 2ybc	Cell parameters from 3936 reflections
*a* = 13.9229 (15) Å	θ = 2.3–25.0°
*b* = 11.6244 (10) Å	µ = 0.83 mm^−^^1^
*c* = 19.553 (2) Å	*T* = 293 K
β = 100.085 (2)°	Needle, red
*V* = 3115.6 (6) Å^3^	0.48 × 0.37 × 0.32 mm
*Z* = 4	

### Data collection

**Table:**

Bruker SMART 1000 CCD area-detector diffractometer	5484 independent reflections
Radiation source: fine-focus sealed tube	3538 reflections with *I* > 2σ(*I*)
graphite	*R*_int_ = 0.032
φ and ω scans	θ_max_ = 25.0°, θ_min_ = 1.5°
Absorption correction: multi-scan (*SADABS*; Bruker, 2001)	*h* = −16→16
*T*_min_ = 0.693, *T*_max_ = 0.778	*k* = −13→13
14599 measured reflections	*l* = −23→13

### Refinement

**Table:**

Refinement on *F*^2^	Primary atom site location: structure-invariant direct methods
Least-squares matrix: full	Secondary atom site location: difference Fourier map
*R*[*F*^2^ > 2σ(*F*^2^)] = 0.042	Hydrogen site location: inferred from neighbouring sites
*wR*(*F*^2^) = 0.117	H-atom parameters constrained
*S* = 1.10	*w* = 1/[σ^2^(*F*_o_^2^) + (0.0402*P*)^2^ + 2.3624*P*] where *P* = (*F*_o_^2^ + 2*F*_c_^2^)/3
5484 reflections	(Δ/σ)_max_ = 0.001
370 parameters	Δρ_max_ = 0.42 e Å^−^^3^
0 restraints	Δρ_min_ = −0.26 e Å^−^^3^

### Special details

**Table:**

Refinement. Refinement of F^2^ against ALL reflections. The weighted *R*-factor *wR* and goodness of fit S are based on F^2^, conventional *R*-factors *R* are based on F, with F set to zero for negative F^2^. The threshold expression of F^2^ > 2sigma(F^2^) is used only for calculating *R*-factors(gt) *etc*. and is not relevant to the choice of reflections for refinement. *R*-factors based on F^2^ are statistically about twice as large as those based on F, and R-factors based on ALL data will be even larger.

### Fractional atomic coordinates and isotropic or equivalent isotropic displacement parameters (Å^2^)

**Table:**

	*x*	*y*	*z*	*U*_iso_*/*U*_eq_	
Ni1	0.77033 (3)	0.40652 (4)	0.63023 (2)	0.04036 (15)	
O1	0.75357 (19)	0.2853 (2)	0.69264 (13)	0.0507 (7)	
O2	0.7291 (2)	0.2026 (3)	0.78879 (16)	0.0759 (9)	
O3	0.8043 (4)	0.5076 (4)	0.3236 (2)	0.1394 (18)	
H3	0.7870	0.4408	0.3155	0.209*	
P1	0.79225 (7)	0.54152 (9)	0.56033 (5)	0.0436 (3)	
P2	0.81015 (7)	0.28485 (8)	0.55286 (5)	0.0411 (2)	
S1	0.74372 (7)	0.54170 (9)	0.70171 (5)	0.0510 (3)	
C1	0.7130 (3)	0.2809 (3)	0.7457 (2)	0.0475 (9)	
C2	0.6393 (3)	0.3695 (3)	0.75551 (18)	0.0438 (9)	
C3	0.6457 (3)	0.4843 (3)	0.73642 (19)	0.0471 (9)	
C4	0.5722 (3)	0.5591 (4)	0.7480 (2)	0.0725 (13)	
H4	0.5763	0.6366	0.7369	0.087*	
C5	0.4931 (4)	0.5208 (5)	0.7755 (3)	0.0915 (17)	
H5	0.4437	0.5718	0.7812	0.110*	
C6	0.4872 (3)	0.4087 (5)	0.7944 (3)	0.0829 (15)	
H6	0.4338	0.3827	0.8126	0.099*	
C7	0.5615 (3)	0.3340 (4)	0.7861 (2)	0.0611 (11)	
H7	0.5593	0.2584	0.8014	0.073*	
C8	0.8596 (3)	0.4831 (3)	0.4959 (2)	0.0527 (10)	
H8A	0.8536	0.5341	0.4561	0.063*	
H8B	0.9282	0.4759	0.5159	0.063*	
C9	0.8175 (3)	0.3661 (3)	0.47359 (19)	0.0496 (10)	
H9A	0.8591	0.3265	0.4463	0.060*	
H9B	0.7531	0.3744	0.4455	0.060*	
C10	0.6782 (3)	0.5982 (3)	0.5114 (2)	0.0523 (10)	
C11	0.6009 (3)	0.6230 (4)	0.5438 (3)	0.0807 (15)	
H11	0.6077	0.6127	0.5916	0.097*	
C12	0.5135 (3)	0.6628 (5)	0.5074 (4)	0.0972 (19)	
H12	0.4629	0.6823	0.5306	0.117*	
C13	0.5019 (4)	0.6734 (5)	0.4376 (4)	0.096 (2)	
H13	0.4422	0.6971	0.4125	0.116*	
C14	0.5748 (5)	0.6504 (5)	0.4049 (3)	0.109 (2)	
H14	0.5660	0.6592	0.3569	0.130*	
C15	0.6655 (4)	0.6127 (5)	0.4412 (3)	0.0929 (18)	
H15	0.7165	0.5978	0.4175	0.111*	
C16	0.8670 (3)	0.6616 (3)	0.59772 (19)	0.0490 (10)	
C17	0.8367 (3)	0.7736 (4)	0.5916 (2)	0.0642 (12)	
H17	0.7744	0.7913	0.5682	0.077*	
C18	0.8993 (4)	0.8608 (4)	0.6203 (3)	0.0812 (15)	
H18	0.8788	0.9370	0.6157	0.097*	
C19	0.9905 (4)	0.8357 (5)	0.6552 (3)	0.0829 (16)	
H19	1.0317	0.8946	0.6745	0.099*	
C20	1.0213 (3)	0.7247 (5)	0.6618 (2)	0.0753 (14)	
H20	1.0837	0.7078	0.6852	0.090*	
C21	0.9599 (3)	0.6372 (4)	0.6338 (2)	0.0642 (12)	
H21	0.9809	0.5613	0.6391	0.077*	
C22	0.9319 (3)	0.2273 (3)	0.58174 (18)	0.0441 (9)	
C23	0.9691 (3)	0.2240 (5)	0.6509 (2)	0.0847 (17)	
H23	0.9337	0.2554	0.6826	0.102*	
C24	1.0588 (4)	0.1746 (5)	0.6744 (3)	0.100 (2)	
H24	1.0824	0.1713	0.7219	0.119*	
C25	1.1126 (3)	0.1313 (4)	0.6301 (3)	0.0783 (15)	
H25	1.1743	0.1016	0.6464	0.094*	
C26	1.0760 (4)	0.1313 (5)	0.5615 (3)	0.105 (2)	
H26	1.1118	0.0991	0.5304	0.125*	
C27	0.9856 (3)	0.1791 (5)	0.5375 (2)	0.0917 (18)	
H27	0.9610	0.1782	0.4901	0.110*	
C28	0.7366 (3)	0.1591 (3)	0.52550 (19)	0.0438 (9)	
C29	0.6959 (4)	0.0986 (4)	0.5731 (2)	0.0695 (13)	
H29	0.7037	0.1234	0.6189	0.083*	
C30	0.6425 (4)	−0.0007 (5)	0.5523 (3)	0.0901 (17)	
H30	0.6144	−0.0416	0.5846	0.108*	
C31	0.6312 (3)	−0.0381 (4)	0.4863 (3)	0.0742 (14)	
H31	0.5960	−0.1051	0.4736	0.089*	
C32	0.6703 (4)	0.0206 (4)	0.4382 (3)	0.0748 (14)	
H32	0.6624	−0.0058	0.3926	0.090*	
C33	0.7224 (3)	0.1206 (4)	0.4574 (2)	0.0672 (13)	
H33	0.7480	0.1623	0.4242	0.081*	
C34	0.8066 (7)	0.5619 (7)	0.2643 (4)	0.192 (4)	
H34A	0.7706	0.6325	0.2633	0.288*	
H34B	0.7778	0.5141	0.2262	0.288*	
H34C	0.8730	0.5784	0.2606	0.288*	

### Atomic displacement parameters (Å^2^)

**Table:**

	*U*^11^	*U*^22^	*U*^33^	*U*^12^	*U*^13^	*U*^23^
Ni1	0.0375 (3)	0.0465 (3)	0.0377 (3)	0.0053 (2)	0.0082 (2)	−0.0006 (2)
O1	0.0646 (17)	0.0471 (16)	0.0446 (15)	0.0164 (13)	0.0210 (14)	0.0023 (12)
O2	0.087 (2)	0.073 (2)	0.074 (2)	0.0221 (18)	0.0314 (18)	0.0313 (18)
O3	0.217 (5)	0.109 (3)	0.090 (3)	−0.041 (3)	0.021 (3)	−0.015 (3)
P1	0.0375 (5)	0.0488 (6)	0.0436 (6)	0.0021 (4)	0.0048 (5)	0.0033 (5)
P2	0.0407 (5)	0.0480 (6)	0.0349 (5)	0.0083 (4)	0.0079 (4)	0.0013 (4)
S1	0.0550 (6)	0.0490 (6)	0.0518 (6)	−0.0048 (5)	0.0168 (5)	−0.0098 (5)
C1	0.044 (2)	0.049 (2)	0.048 (2)	−0.0002 (18)	0.0054 (19)	−0.002 (2)
C2	0.040 (2)	0.057 (3)	0.034 (2)	−0.0029 (18)	0.0049 (17)	−0.0051 (18)
C3	0.045 (2)	0.056 (3)	0.041 (2)	0.0072 (19)	0.0099 (18)	−0.0073 (19)
C4	0.076 (3)	0.070 (3)	0.077 (3)	0.026 (3)	0.030 (3)	0.001 (3)
C5	0.070 (3)	0.108 (5)	0.106 (4)	0.032 (3)	0.042 (3)	0.002 (4)
C6	0.059 (3)	0.109 (5)	0.089 (4)	−0.003 (3)	0.035 (3)	−0.007 (3)
C7	0.058 (3)	0.071 (3)	0.058 (3)	−0.006 (2)	0.019 (2)	−0.006 (2)
C8	0.047 (2)	0.064 (3)	0.048 (2)	0.006 (2)	0.0114 (19)	0.010 (2)
C9	0.042 (2)	0.068 (3)	0.040 (2)	0.0117 (19)	0.0102 (17)	0.0093 (19)
C10	0.047 (2)	0.047 (2)	0.058 (3)	0.0043 (19)	−0.005 (2)	0.001 (2)
C11	0.047 (3)	0.103 (4)	0.091 (4)	0.011 (3)	0.007 (3)	0.029 (3)
C12	0.043 (3)	0.110 (5)	0.134 (5)	0.009 (3)	0.003 (3)	0.032 (4)
C13	0.069 (4)	0.080 (4)	0.120 (5)	0.011 (3)	−0.037 (4)	−0.013 (4)
C14	0.119 (5)	0.120 (5)	0.070 (4)	0.053 (4)	−0.033 (4)	−0.012 (3)
C15	0.096 (4)	0.112 (4)	0.064 (3)	0.045 (3)	−0.004 (3)	−0.003 (3)
C16	0.046 (2)	0.056 (3)	0.046 (2)	−0.0077 (19)	0.0098 (19)	0.001 (2)
C17	0.073 (3)	0.055 (3)	0.064 (3)	−0.005 (2)	0.009 (2)	0.006 (2)
C18	0.108 (4)	0.054 (3)	0.084 (4)	−0.017 (3)	0.026 (3)	−0.008 (3)
C19	0.087 (4)	0.097 (4)	0.068 (3)	−0.041 (3)	0.023 (3)	−0.014 (3)
C20	0.058 (3)	0.102 (4)	0.064 (3)	−0.021 (3)	0.008 (2)	−0.009 (3)
C21	0.053 (3)	0.075 (3)	0.063 (3)	−0.007 (2)	0.006 (2)	−0.003 (2)
C22	0.042 (2)	0.051 (2)	0.040 (2)	0.0077 (17)	0.0103 (17)	0.0026 (18)
C23	0.062 (3)	0.142 (5)	0.048 (3)	0.045 (3)	0.003 (2)	−0.013 (3)
C24	0.074 (3)	0.162 (6)	0.057 (3)	0.055 (4)	−0.004 (3)	−0.007 (3)
C25	0.053 (3)	0.110 (4)	0.070 (3)	0.028 (3)	0.005 (3)	0.014 (3)
C26	0.087 (4)	0.167 (6)	0.067 (3)	0.073 (4)	0.034 (3)	0.023 (4)
C27	0.080 (3)	0.151 (5)	0.046 (3)	0.061 (4)	0.014 (2)	0.010 (3)
C28	0.040 (2)	0.049 (2)	0.042 (2)	0.0079 (18)	0.0068 (17)	0.0008 (19)
C29	0.096 (4)	0.062 (3)	0.054 (3)	−0.010 (3)	0.023 (3)	−0.004 (2)
C30	0.122 (5)	0.077 (4)	0.078 (4)	−0.033 (3)	0.038 (3)	0.002 (3)
C31	0.072 (3)	0.058 (3)	0.088 (4)	−0.010 (2)	0.004 (3)	−0.003 (3)
C32	0.082 (3)	0.071 (3)	0.066 (3)	−0.011 (3)	−0.003 (3)	−0.013 (3)
C33	0.075 (3)	0.074 (3)	0.053 (3)	−0.010 (3)	0.011 (2)	−0.003 (2)
C34	0.264 (11)	0.185 (9)	0.123 (6)	−0.099 (8)	0.025 (7)	0.050 (6)

### Geometric parameters (Å, °)

**Table:**

Ni1—O1	1.905 (3)	C14—C15	1.406 (7)
Ni1—P1	2.1378 (11)	C14—H14	0.9300
Ni1—S1	2.1775 (11)	C15—H15	0.9300
Ni1—P2	2.2114 (10)	C16—C17	1.367 (5)
O1—C1	1.267 (4)	C16—C21	1.390 (5)
O2—C1	1.234 (4)	C17—C18	1.390 (6)
O3—C34	1.326 (7)	C17—H17	0.9300
O3—H3	0.8200	C18—C19	1.363 (7)
P1—C16	1.817 (4)	C18—H18	0.9300
P1—C10	1.827 (4)	C19—C20	1.358 (7)
P1—C8	1.828 (4)	C19—H19	0.9300
P2—C28	1.811 (4)	C20—C21	1.378 (6)
P2—C22	1.818 (4)	C20—H20	0.9300
P2—C9	1.833 (4)	C21—H21	0.9300
S1—C3	1.759 (4)	C22—C27	1.360 (5)
C1—C2	1.490 (5)	C22—C23	1.362 (5)
C2—C7	1.389 (5)	C23—C24	1.378 (6)
C2—C3	1.393 (5)	C23—H23	0.9300
C3—C4	1.391 (5)	C24—C25	1.339 (6)
C4—C5	1.381 (6)	C24—H24	0.9300
C4—H4	0.9300	C25—C26	1.348 (6)
C5—C6	1.360 (7)	C25—H25	0.9300
C5—H5	0.9300	C26—C27	1.381 (6)
C6—C7	1.381 (6)	C26—H26	0.9300
C6—H6	0.9300	C27—H27	0.9300
C7—H7	0.9300	C28—C29	1.366 (5)
C8—C9	1.514 (5)	C28—C33	1.386 (5)
C8—H8A	0.9700	C29—C30	1.394 (6)
C8—H8B	0.9700	C29—H29	0.9300
C9—H9A	0.9700	C30—C31	1.345 (7)
C9—H9B	0.9700	C30—H30	0.9300
C10—C15	1.363 (6)	C31—C32	1.352 (6)
C10—C11	1.372 (6)	C31—H31	0.9300
C11—C12	1.379 (6)	C32—C33	1.387 (6)
C11—H11	0.9300	C32—H32	0.9300
C12—C13	1.351 (8)	C33—H33	0.9300
C12—H12	0.9300	C34—H34A	0.9600
C13—C14	1.318 (8)	C34—H34B	0.9600
C13—H13	0.9300	C34—H34C	0.9600
			
O1—Ni1—P1	178.81 (8)	C13—C14—C15	121.1 (6)
O1—Ni1—S1	93.94 (8)	C13—C14—H14	119.4
P1—Ni1—S1	86.56 (4)	C15—C14—H14	119.4
O1—Ni1—P2	92.22 (8)	C10—C15—C14	119.6 (5)
P1—Ni1—P2	87.20 (4)	C10—C15—H15	120.2
S1—Ni1—P2	172.70 (4)	C14—C15—H15	120.2
C1—O1—Ni1	132.6 (3)	C17—C16—C21	119.0 (4)
C34—O3—H3	109.5	C17—C16—P1	123.4 (3)
C16—P1—C10	108.55 (18)	C21—C16—P1	117.7 (3)
C16—P1—C8	103.64 (18)	C16—C17—C18	119.8 (4)
C10—P1—C8	106.08 (19)	C16—C17—H17	120.1
C16—P1—Ni1	116.27 (13)	C18—C17—H17	120.1
C10—P1—Ni1	113.02 (14)	C19—C18—C17	120.6 (5)
C8—P1—Ni1	108.39 (13)	C19—C18—H18	119.7
C28—P2—C22	104.04 (17)	C17—C18—H18	119.7
C28—P2—C9	106.18 (18)	C20—C19—C18	120.1 (5)
C22—P2—C9	105.40 (17)	C20—C19—H19	119.9
C28—P2—Ni1	121.47 (12)	C18—C19—H19	119.9
C22—P2—Ni1	110.78 (12)	C19—C20—C21	120.0 (5)
C9—P2—Ni1	107.84 (13)	C19—C20—H20	120.0
C3—S1—Ni1	101.81 (13)	C21—C20—H20	120.0
O2—C1—O1	122.4 (4)	C20—C21—C16	120.5 (5)
O2—C1—C2	118.0 (4)	C20—C21—H21	119.7
O1—C1—C2	119.6 (4)	C16—C21—H21	119.7
C7—C2—C3	119.5 (4)	C27—C22—C23	117.7 (4)
C7—C2—C1	117.2 (4)	C27—C22—P2	122.7 (3)
C3—C2—C1	123.4 (3)	C23—C22—P2	119.4 (3)
C4—C3—C2	118.2 (4)	C22—C23—C24	120.5 (4)
C4—C3—S1	117.9 (3)	C22—C23—H23	119.8
C2—C3—S1	123.9 (3)	C24—C23—H23	119.8
C5—C4—C3	121.3 (5)	C25—C24—C23	121.2 (5)
C5—C4—H4	119.3	C25—C24—H24	119.4
C3—C4—H4	119.3	C23—C24—H24	119.4
C6—C5—C4	120.3 (5)	C24—C25—C26	119.2 (4)
C6—C5—H5	119.8	C24—C25—H25	120.4
C4—C5—H5	119.8	C26—C25—H25	120.4
C5—C6—C7	119.3 (5)	C25—C26—C27	120.1 (5)
C5—C6—H6	120.4	C25—C26—H26	120.0
C7—C6—H6	120.4	C27—C26—H26	120.0
C6—C7—C2	121.3 (4)	C22—C27—C26	121.3 (4)
C6—C7—H7	119.4	C22—C27—H27	119.4
C2—C7—H7	119.4	C26—C27—H27	119.4
C9—C8—P1	108.2 (3)	C29—C28—C33	118.8 (4)
C9—C8—H8A	110.1	C29—C28—P2	119.7 (3)
P1—C8—H8A	110.1	C33—C28—P2	121.5 (3)
C9—C8—H8B	110.1	C28—C29—C30	119.4 (4)
P1—C8—H8B	110.1	C28—C29—H29	120.3
H8A—C8—H8B	108.4	C30—C29—H29	120.3
C8—C9—P2	107.2 (3)	C31—C30—C29	121.0 (5)
C8—C9—H9A	110.3	C31—C30—H30	119.5
P2—C9—H9A	110.3	C29—C30—H30	119.5
C8—C9—H9B	110.3	C30—C31—C32	120.7 (5)
P2—C9—H9B	110.3	C30—C31—H31	119.7
H9A—C9—H9B	108.5	C32—C31—H31	119.7
C15—C10—C11	117.7 (4)	C31—C32—C33	119.4 (5)
C15—C10—P1	121.3 (4)	C31—C32—H32	120.3
C11—C10—P1	120.9 (3)	C33—C32—H32	120.3
C10—C11—C12	121.6 (5)	C28—C33—C32	120.7 (4)
C10—C11—H11	119.2	C28—C33—H33	119.6
C12—C11—H11	119.2	C32—C33—H33	119.6
C13—C12—C11	119.4 (6)	O3—C34—H34A	109.5
C13—C12—H12	120.3	O3—C34—H34B	109.5
C11—C12—H12	120.3	H34A—C34—H34B	109.5
C14—C13—C12	120.4 (5)	O3—C34—H34C	109.5
C14—C13—H13	119.8	H34A—C34—H34C	109.5
C12—C13—H13	119.8	H34B—C34—H34C	109.5
			
P1—Ni1—O1—C1	134 (4)	C16—P1—C10—C11	−85.0 (4)
S1—Ni1—O1—C1	19.1 (3)	C8—P1—C10—C11	164.1 (4)
P2—Ni1—O1—C1	−164.8 (3)	Ni1—P1—C10—C11	45.5 (4)
O1—Ni1—P1—C16	−70 (4)	C15—C10—C11—C12	−0.9 (8)
S1—Ni1—P1—C16	44.69 (15)	P1—C10—C11—C12	−178.2 (4)
P2—Ni1—P1—C16	−131.52 (15)	C10—C11—C12—C13	2.7 (9)
O1—Ni1—P1—C10	163 (4)	C11—C12—C13—C14	−2.8 (9)
S1—Ni1—P1—C10	−81.83 (15)	C12—C13—C14—C15	1.0 (10)
P2—Ni1—P1—C10	101.96 (15)	C11—C10—C15—C14	−0.9 (8)
O1—Ni1—P1—C8	46 (4)	P1—C10—C15—C14	176.4 (4)
S1—Ni1—P1—C8	160.88 (14)	C13—C14—C15—C10	0.9 (9)
P2—Ni1—P1—C8	−15.33 (14)	C10—P1—C16—C17	3.2 (4)
O1—Ni1—P2—C28	49.42 (16)	C8—P1—C16—C17	115.6 (4)
P1—Ni1—P2—C28	−131.62 (14)	Ni1—P1—C16—C17	−125.5 (3)
S1—Ni1—P2—C28	−162.9 (3)	C10—P1—C16—C21	−176.3 (3)
O1—Ni1—P2—C22	−73.03 (15)	C8—P1—C16—C21	−63.8 (3)
P1—Ni1—P2—C22	105.93 (13)	Ni1—P1—C16—C21	55.0 (3)
S1—Ni1—P2—C22	74.6 (4)	C21—C16—C17—C18	1.0 (6)
O1—Ni1—P2—C9	172.11 (15)	P1—C16—C17—C18	−178.4 (3)
P1—Ni1—P2—C9	−8.93 (13)	C16—C17—C18—C19	−0.6 (7)
S1—Ni1—P2—C9	−40.2 (4)	C17—C18—C19—C20	0.4 (8)
O1—Ni1—S1—C3	−41.42 (15)	C18—C19—C20—C21	−0.7 (8)
P1—Ni1—S1—C3	139.66 (13)	C19—C20—C21—C16	1.1 (7)
P2—Ni1—S1—C3	171.0 (3)	C17—C16—C21—C20	−1.2 (6)
Ni1—O1—C1—O2	−162.4 (3)	P1—C16—C21—C20	178.2 (3)
Ni1—O1—C1—C2	20.7 (5)	C28—P2—C22—C27	67.9 (4)
O2—C1—C2—C7	−32.1 (5)	C9—P2—C22—C27	−43.6 (5)
O1—C1—C2—C7	144.9 (4)	Ni1—P2—C22—C27	−160.0 (4)
O2—C1—C2—C3	147.1 (4)	C28—P2—C22—C23	−107.1 (4)
O1—C1—C2—C3	−35.9 (5)	C9—P2—C22—C23	141.4 (4)
C7—C2—C3—C4	−1.0 (6)	Ni1—P2—C22—C23	25.0 (4)
C1—C2—C3—C4	179.8 (4)	C27—C22—C23—C24	0.8 (8)
C7—C2—C3—S1	176.4 (3)	P2—C22—C23—C24	176.1 (5)
C1—C2—C3—S1	−2.8 (5)	C22—C23—C24—C25	1.7 (10)
Ni1—S1—C3—C4	−141.0 (3)	C23—C24—C25—C26	−3.2 (10)
Ni1—S1—C3—C2	41.6 (3)	C24—C25—C26—C27	2.2 (10)
C2—C3—C4—C5	−2.0 (7)	C23—C22—C27—C26	−1.8 (8)
S1—C3—C4—C5	−179.6 (4)	P2—C22—C27—C26	−176.9 (5)
C3—C4—C5—C6	2.3 (8)	C25—C26—C27—C22	0.3 (10)
C4—C5—C6—C7	0.5 (8)	C22—P2—C28—C29	86.9 (4)
C5—C6—C7—C2	−3.6 (7)	C9—P2—C28—C29	−162.1 (3)
C3—C2—C7—C6	3.8 (6)	Ni1—P2—C28—C29	−38.7 (4)
C1—C2—C7—C6	−177.0 (4)	C22—P2—C28—C33	−91.5 (4)
C16—P1—C8—C9	166.6 (3)	C9—P2—C28—C33	19.4 (4)
C10—P1—C8—C9	−79.2 (3)	Ni1—P2—C28—C33	142.9 (3)
Ni1—P1—C8—C9	42.5 (3)	C33—C28—C29—C30	0.9 (7)
P1—C8—C9—P2	−49.1 (3)	P2—C28—C29—C30	−177.5 (4)
C28—P2—C9—C8	168.2 (2)	C28—C29—C30—C31	0.5 (8)
C22—P2—C9—C8	−81.8 (3)	C29—C30—C31—C32	−0.8 (9)
Ni1—P2—C9—C8	36.5 (3)	C30—C31—C32—C33	−0.2 (8)
C16—P1—C10—C15	97.7 (4)	C29—C28—C33—C32	−2.0 (6)
C8—P1—C10—C15	−13.1 (4)	P2—C28—C33—C32	176.5 (3)
Ni1—P1—C10—C15	−131.7 (4)	C31—C32—C33—C28	1.6 (7)

### Hydrogen-bond geometry (Å, °)

**Table:**

*D*—H···*A*	*D*—H	H···*A*	*D*···*A*	*D*—H···*A*
O3—H3···O2^i^	0.82	1.88	2.697 (5)	171

Symmetry codes: (i) *x*, −*y*+1/2, *z*−1/2.
